# Machine learning predictive model for aspiration screening in hospitalized patients with acute stroke

**DOI:** 10.1038/s41598-023-34999-8

**Published:** 2023-05-15

**Authors:** Dougho Park, Seok Il Son, Min Sol Kim, Tae Yeon Kim, Jun Hwa Choi, Sang-Eok Lee, Daeyoung Hong, Mun-Chul Kim

**Affiliations:** 1 Department of Medical Science and Engineering, School of Convergence Science and Technology, Pohang University of Science and Technology, Pohang, Republic of Korea; 2 Department of Rehabilitation Medicine, Pohang Stroke and Spine Hospital, Pohang, Republic of Korea; 3 Occupational Therapy Department of Rehabilitation Center, Pohang Stroke and Spine Hospital, Pohang, Republic of Korea; 4 Speech-Language Therapy Department of Rehabilitation Center, Pohang Stroke and Spine Hospital, Pohang, Republic of Korea; 5 Department of Quality Improvement, Pohang Stroke and Spine Hospital, Pohang, Republic of Korea; 6 Department of Neurosurgery, Pohang Stroke and Spine Hospital, Pohang, Republic of Korea

**Keywords:** Machine learning, Stroke

## Abstract

Dysphagia is a fatal condition after acute stroke. We established machine learning (ML) models for screening aspiration in patients with acute stroke. This retrospective study enrolled patients with acute stroke admitted to a cerebrovascular specialty hospital between January 2016 and June 2022. A videofluoroscopic swallowing study (VFSS) confirmed aspiration. We evaluated the Gugging Swallowing Screen (GUSS), an early assessment tool for dysphagia, in all patients and compared its predictive value with ML models. Following ML algorithms were applied: regularized logistic regressions (ridge, lasso, and elastic net), random forest, extreme gradient boosting, support vector machines, *k*-nearest neighbors, and naïve Bayes. We finally analyzed data from 3408 patients, and 448 of them had aspiration on VFSS. The GUSS showed an area under the receiver operating characteristics curve (AUROC) of 0.79 (0.77–0.81). The ridge regression model was the best model among all ML models, with an AUROC of 0.81 (0.76–0.86), an F1 measure of 0.45. Regularized logistic regression models exhibited higher sensitivity (0.66–0.72) than the GUSS (0.64). Feature importance analyses revealed that the modified Rankin scale was the most important feature of ML performance. The proposed ML prediction models are valid and practical for screening aspiration in patients with acute stroke.

## Introduction

Dysphagia is a common comorbidity after acute stroke^1^, occurring in more than half of the stroke survivors^2^. Moreover, dysphagia associated with acute stroke causes aspiration in many cases, which can result in severe complications such as aspiration pneumonia, dehydration, and malnutrition^3^. Post-stroke pneumonia occurs in about 15% of patients with acute stroke and is a fatal conditions with a 30-day mortality rate of up to 30%^4,5^. Furthermore, it has been reported that up to 40% of patients with acute stroke are at risk of malnutrition, which is linked to pressure ulcers, increased dependency, prolonged institutionalization, and high mortality rates^6,7^. Moreover, aspiration increases the burden of the initial medical treatment, and active rehabilitation and return to society are inevitably delayed. As a result, the patient’s long-term prognosis is adversely affected due to dysphagia and aspiration, which in turn causes a vicious cycle that deteriorates the patient’s quality of life^8^.

Therefore, early screening for dysphagia and aspiration develops an appropriate feeding strategy^9^. The Gugging Swallowing Screen (GUSS), introduced by Trapl et al.^10^, is one of the most widely used dysphagia screening tools in the clinical field and has undergone the most validation testing^11,12^. The GUSS comprises direct and indirect evaluations. The indirect test assesses dry saliva swallowing, level of consciousness, and the ability to cough. The direct test evaluates signs of swallowing difficulties, such as delayed swallowing, coughing, drooling, and voice changes after food intake^13^. Although the GUSS has the advantage of being relatively easy to perform at the bedside, its direct evaluation is invasive and carries aspiration pneumonia risk in patients with acute stroke. In addition, the examiner should be sufficiently trained to ensure the reliability of the results. Further, the direct swallowing test sometimes has a high false-positive rate, leading to unnecessary referrals for further testing^14^. Finally, accurate evaluation is limited in patients with cognitive impairment or communication difficulties^15^. There are questionnaires such as the dysphagia handicap index and eating assessment tool-10 to screen dysphagia early in hospitalization^16,17^; they are widely used because of their easy-to-perform and non-invasive advantages and have been translated into various languages and passed through many validation tests. However, they also have a fundamental disadvantage because they can be applied only to a limited patient group. Moreover, some screening tools not only lack standardization in terms of the timing and frequency of examination but also have not been adequately validated in diverse patient populations or settings^18^. For these reasons, studies reporting on the predictive power of dysphagia screening tools were often heterogenous and did not provide precisely estimated results^19^.

The videofluoroscopic swallowing study (VFSS) has been widely used as a confirmatory test to diagnose aspiration^20^; despite the advantage of accurately determining whether aspiration is achieved through visualization of all stages of swallowing, it is still invasive and has the disadvantage of being exposed to radiation^21^. In addition, the examination C-arm device is required, and the test is possible in a state where the patient can maintain an appropriate posture^22^. VFSS also needs suitable space and schedule for these two limitations. Therefore, it can be considered that the VFSS is inappropriate for screening patients with acute stroke.

Machine learning (ML) algorithms are good at performing regression and classification by learning from tubular data^23^. Some parts can be a bit confusing when you come across the term “learning,” but it would be correct to say that ML algorithms perform calculations rather than learning. The generally used method in current ML-related medical research has been supervised learning, which compares the real-world output that comes out through a human expert’s decision with the result calculated by ML algorithms based on the same input data^24,25^. Based on electrical health records (EHRs), ML models have been widely proposed for the diagnosis, treatment, and prognosis of diseases^26,27^. In particular, ML models for the early detection of diseases have been presented in various fields, such as coronary heart disease^28^, aortic dissections^29^, depression^30^, and Alzheimer’s disease^31^, and the results have demonstrated that ML prediction models are comparable to existing screening tools. In addition, ML algorithms have the advantage of using EHR to develop predictive models relatively readily and efficiently, even using a dataset consisting of a large sample with numerous variables. However, despite the rapid expansion of medical research applying ML algorithms, to the best of our knowledge, no reports have presented an ML-based model for screening aspiration in patients with acute stroke. Although Jauk et al.^32^ introduced the ML-based dysphagia prediction model, which showed acceptable prediction performance in the geriatric cohort, no study targeted patients with acute stroke using ML-based aspiration prediction models.

This study aimed to establish ML prediction models to screen for aspiration, confirmed by VFSS, particularly applicable for patients with acute stroke. According to the screening tool’s purpose, the initial information obtained before the VFSS was used as potential predictors and compared the performance of ML models with the predictive power of the GUSS as a traditional screening tool. To explain the causality of variables, we used both the stepwise logistic regression model and the feature importance analysis of ML models. Ultimately, this study investigated whether ML prediction models enabled early and accurate aspiration screening after acute stroke and could be a reliable alternative to traditional aspiration screening.

## Methods

### Study population and ethical statements

**Figure 1:**
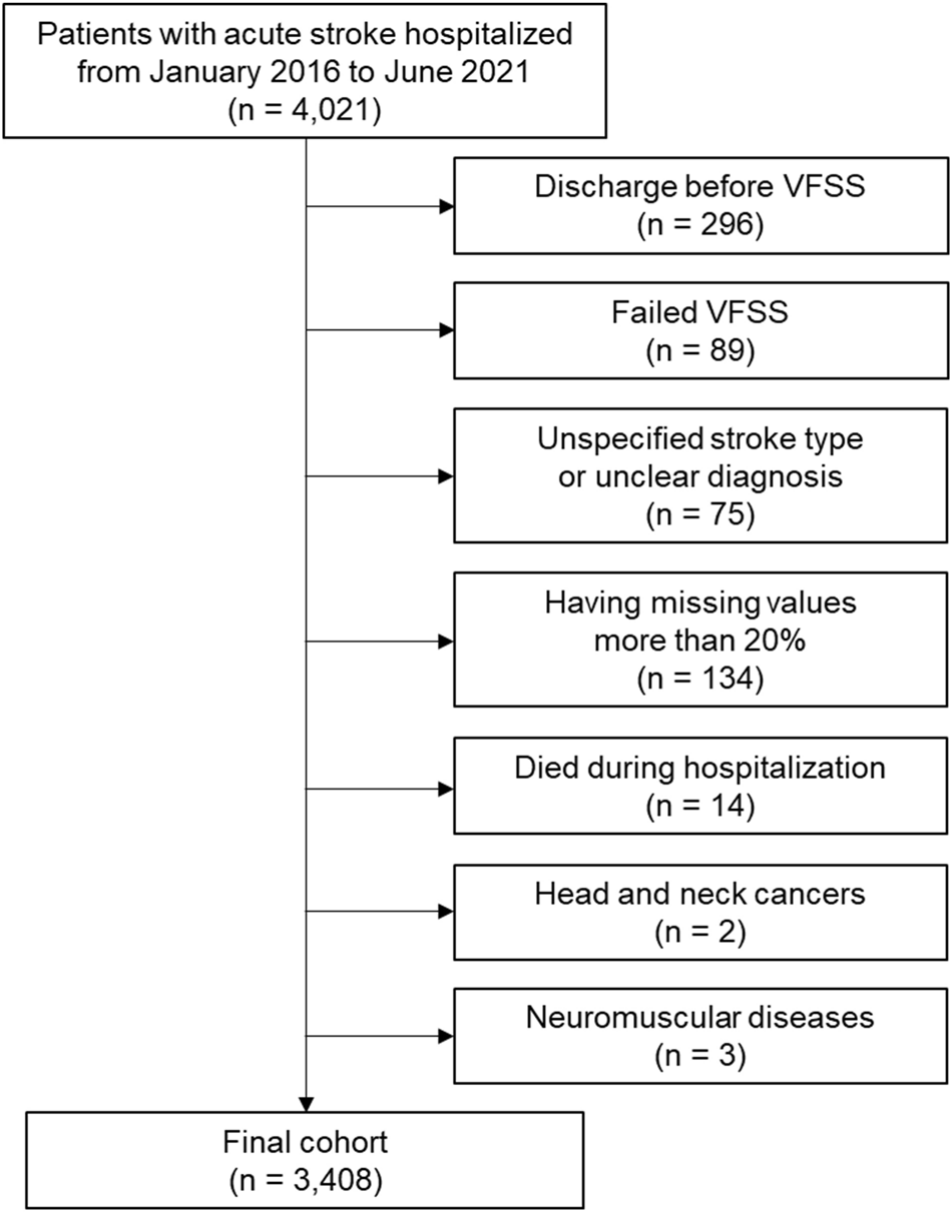
Flowchart of patient inclusion. *VFSS* videofluoroscopic swallowing study.

This retrospective study utilized EHRs of patients hospitalized with acute stroke between January 2016 and June 2022 at a single cerebrovascular specialty hospital. Acute stroke was defined as hospitalization within seven days of new-onset stroke, and patients hospitalized with International Classification of Diseases-10 codes of I60–I63 were selected. The following exclusion criteria were applied in this study: (1) discharged before completion of the VFSS, (2) VFSS failed because of poor cooperation, (3) unspecified stroke type or unclear diagnosis, (4) missing values or lack of clinical information (more than 20%), (5) mortality during the hospitalization period, (6) head and neck cancers, (7) neuromuscular diseases, and (8) underwent prior radiation therapy (Fig. 1). The institutional review board of Pohang Stroke and Spine Hospital reviewed and approved the study design (PSSH0475-202201-HR-001-01). All data was anonymized, excluding patients’ resident and hospital registration numbers and detailed home addresses. Then the dataset was exported to the authorized researcher for this study. Informed consent was waived owing to the study’s retrospective nature by the institutional review board of Pohang Stroke and Spine Hospital. This study was conducted in compliance with the Declaration of Helsinki and the International Conference on Harmonization–Good Clinical Practice Guidelines.

### The rationale for selecting the potential predictors

We applied the following criteria in extracting potential predictors. First, personal and clinical information available within a short time after admission was defined as potential predictors to develop an ML prediction model for screening purposes. Second, as much as possible, the variables identified as risk factors for post-stroke dysphagia or pneumonia reported in previous studies were included. Thirdly, the variable had to be reliably evaluated and readily extracted from EHR, and the missing value should not exceed 20% of the total.

Age and sex have been known risk factors for stroke and stroke-related pneumonia^33^. Meanwhile, smoking, obesity, and comorbidities such as hypertension, diabetes, dyslipidemia, and previous cerebrovascular lesions, which act as vascular risk factors, are also significant variables that increase the risk of aspiration after stroke^34,35^. Symptoms related to motor impairment, such as impaired physical morbidity and dysarthria, are also known to increase the risk of stroke-related aspiration^36^. Among stroke-related factors, it has been known that the higher frequency of dysphagia was associated with brain stem lesions, hemorrhagic stroke, and stroke severity^37^. Additionally, malnutrition is a complication of dysphagia and increases the risk of stroke-related aspiration^38^.

Basic patient information, including age, sex, and socioeconomic status, was identified first based on these previous reports. Then, to confirm the nutritional state and general condition of the patient with acute stroke, mental status, vital signs, and laboratory findings at admission were investigated. In addition, patient or guardian interviews, medical records, and medication history were used to identify the patient’s comorbidities. As stroke-related factors, stroke type and territories were identified. Further, the modified Rankin scale, Morse Fall scale, facial asymmetry, and aphasia were checked to identify the motor and functional impairments. Detailed definitions of each variable are presented in Supplementary Table S1.

Patients with acute stroke underwent GUSS examinations when consultation with early rehabilitation was received; this mainly occurred before the VFSS. Skilled occupational therapists performed the GUSS. The highest score on the GUSS is 20; the higher score means less severe swallowing difficulties.

### Videofluoroscopic swallowing study and outcome definition

We used a ZEN-5000 C-arm fluoroscope for the VFSS (Genoray Inc., Seongnam, Korea). The patient maintained an upright sitting posture in a chair or wheelchair, and postural support was provided if the patient could not sit upright. As a contrast agent, 230% barium liquid was diluted to approximately 35% in free water. Food forms consisted of solid, semi-solid, and liquid (2 ml, 5 ml, and 90 ml, respectively). Three examiners from a multidisciplinary team performed the VFSS and on-site interpretations. The team consisted of rehabilitation medicine specialists, occupational therapists, and a speech-language therapist. The next day, the same team reviewed the video recording again for an accurate interpretation. Interpretations were primarily based on the patient’s sagittal view images. We defined aspiration, the target outcome of this study, as the detection of one or more swallowing with a Penetration-Aspiration scale score of 6–8 on the VFSS during hospitalization^39^.

### Statistical analysis

Statistical analyses were performed using R software version 4.2.3 (R Core Team, R Foundation for Statistical Computing, Vienna, Austria). Continuous variables were tested for normality using the Shapiro–Wilk test and are expressed as median (interquartile range). The Wilcoxon rank-sum test was then applied for comparative analysis between the two groups. Categorical variables are expressed as frequency (proportion). The chi-squared (trend) test was used for comparative analysis between the two groups. The area under the receiver operating characteristic curve (AUROC) was analyzed using the “Epi” package in the R software to determine the predictive value of the GUSS for aspiration^40^. We established a stepwise logistic regression model using the backward elimination method to interpret the adjusted odds ratio (aOR) for predicting aspiration. During the stepwise elimination of covariates, the model fitness was assessed using the Akaike information criterion. Multicollinearity between variables was confirmed using the variation inflation factor, with sqrt (variation inflation factor) > 2 as the threshold. We defined statistical significance as a *P*-value less than 0.05.

### Machine learning

#### Data pre-processing and model establishing

We used the “caret” package of the R software for the ML modeling process^41^. Before ML modeling, the data were pre-processed. First, we identified variables with near-zero-variance and removed them. Then, the threshold was set at a correlation coefficient > 0.7 to check for multicollinearity between continuous variables. Continuous variables were then subjected to centering and scaling. Categorical variables underwent one-hot encoding and were transformed into dummy variables. We also detected and removed variables and individuals with more than 20% missing values. Then, we imputed remained missing values while applying a multivariate imputation via the method of the chained equation^42^.

**Figure 2:**
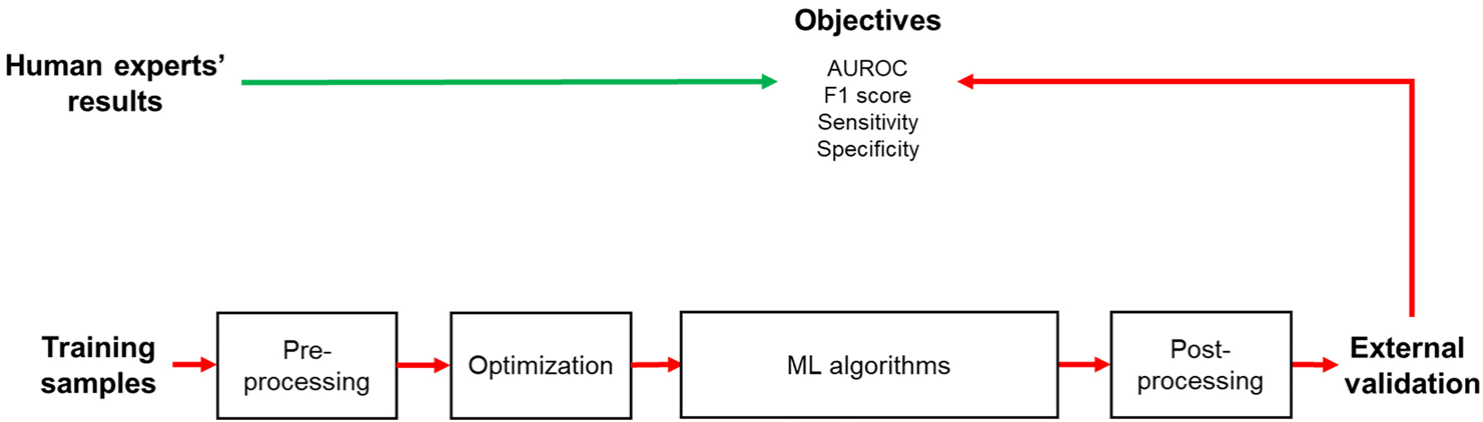
Schematic diagram of the machine learning process in this study. *AUROC* area under the receiver operating characteristics curve.

We randomly allocated the entire data into 75% of the training set and 25% of the test set for ML prediction. A synthetic minority oversampling technique was applied to balance the target classes of the training dataset. We utilized the following ML algorithms to generate the prediction model: regularized logistic regression (RLRs)–ridge, lasso, and elastic net–and ensemble algorithms such as random forest (RF) and extreme gradient boosting (XGB). We also utilized classic ML classifiers such as support vector machines (SVM), *k*-nearest neighbors (KNN), and naïve Bayes (NB). We performed five-fold cross-validation with 50 repeats for an optimal training model. In addition, we used a random or grid search for hyperparameter tuning. We provide tuned hyperparameters and their searching method for each model in Supplementary Table S2. The AUROC, F1 score, sensitivity, and specificity were used as metrics to measure the performance of the ML models (Fig. 2). The entire code for the machine learning process is available in the Online Supplementary Content S1.

#### Regularized logistic regressions

Some classical algorithms have the advantage of being fast and easy to apply, but the biggest problem is the possibility of overfitting^43^. Overfitting is defined as when the data has many features, and the hypothesis function fits nicely on the training data. However, it fails to generalize the validation data, a common problem when doing ML modeling^44^. Logistic regression is based on a linear model commonly used in medical statistics, and the more features it has, the more vulnerable it is to overfitting. RLR proceeds in a way that minimizes overfitting through regularization; it overcomes overfitting with a non-sparse solution (L2 regularization, Ridge) or sparse solution method (L1 regularization, Lasso) for high-order variables in a linear equation while maximizing predictive power^45^. Meanwhile, the elastic net method performs regularization by the hybrid method of Ridge and Lasso^46^.

#### Ensemble algorithms

Ensemble learning is a technique for deriving more accurate results by creating multiple classifiers and combining the predictions. This method helps more accurate prediction by combining several weaker models instead of one robust model, and bagging and boosting types are the most representative^47,48^.

RF is a representative ensemble algorithm that uses a bagging method based on a decision tree^49^. It is an algorithm that improves predictive power while solving the overfitting problem that inevitably occurs as the number of branches in the decision tree increases^50^. This algorithm allows duplication of data division during the bagging process, and through this, a unique dataset can be continuously formed^51^.

The boosting method differs from bagging in that several classifiers perform learning sequentially, and predictions are performed while weighting the next classifier^52^. One representative boosting module is XGB, which provides optimized custom options by providing parallel processing techniques and various hyperparameter settings^53^. Therefore, it solves the problem of slow process and overfitting of the boosting method with sequential features in general and shows high predictability simultaneously^54^.

#### Other classic classifiers

SVM is a classical ML algorithm that creates a virtual vector space; then, it finds the margins that separate each group and recognizes patterns based on such boundaries^55^. SVM performs the classification task by maximizing the distance between the margins that classify the two groups^56^. SVM can be used not only for linear classification but also for non-linear classification through kernelization. On the other hand, SVM is not suited for datasets with a lot of noise^57^.

KNN works by finding the nearest neighbors based on the distance between data points and predicting the label of new data by referring to the labels of those neighbors^58^. KNN measures the distance between all data points each time, so the computation cost is high. However, it has the advantage of obtaining simple and good classification performance when the data is relatively small^59^.

Bayes' theorem is a formula that calculates conditional probability, which is the probability of an event occurring, given that another event has already happened^60^. Based on Bayes' theorem, NB calculates the probability that the input data belongs to each class. Like other classic classifiers, NB has the advantage of fast model learning and efficient data processing^61^. On the other hand, NB is calculated based on the assumption that each feature is independent. Therefore, accuracy may be low if some features' independence assumption is unsuitable^62^.

## Results

### Baseline characteristics

A total of 3408 hospitalized patients with acute stroke were included for analysis. Among them, 448 patients presented with aspiration on VFSS during hospitalization. The results of the baseline characteristics and comparison analyses between the aspiration and non-aspiration groups are presented in Table 1. The aspiration group was significantly older than the non-aspiration group (73.0 [63.0–79.0] vs. 67.0 [58.0–77.0] years old; *p* < 0.001). Furthermore, the ratio of males, medical aid, previous cerebrovascular accidents, and diabetes were significantly higher in the aspiration group (*p* = 0.002, *p* < 0.001, *p* < 0.001, and *p* = 0.036, respectively). In addition, the ratio of dyslipidemia was significantly lower in the aspiration group (*p* = 0.013). Among the stroke-related features, the aspiration group had a significantly higher rate of hemorrhagic stroke (25.9% *vs.* 18.5%; *p* < 0.001), initially altered mental status (29.5% *vs.* 6.3%; *p* < 0.001), aphasia (18.1% *vs.* 5.5%; *p* < 0.001), and facial asymmetry (63.4% *vs.* 40.0%; *p* < 0.001) than the non-aspiration group. Additionally, the aspiration group showed a significantly higher rate of patients admitted via the emergency department (91.1% *vs.* 86.9%; *p* = 0.016) and more severe functional deterioration–higher modified Rankin scale and Morse Fall scale (3.0 [2.0–4.0] *vs.* 2.0 [1.0–3.0]; *p* < 0.001 and 35.0 [35.0–50.0] *vs.* 35.0 [20.0–35.0]; *p* < 0.001, respectively). Finally, days from admission to the initial VFSS study were significantly longer in the aspiration group than in the non-aspiration group (5.0 [3.0–8.0] *vs.* 2.0 [1.0–5.0] days; *p* < 0.001).

**Table 1:** Baseline characteristics.

Variables	No aspiration (n = 2,960)	Aspiration (n = 448)	*p*-value
Age, years	67.0 (58.0–77.0)	73.0 (63.0–79.0)	< 0.001
Male, n (%)	1735 (58.6)	297 (66.3)	0.002
Body mass index, kg/m^2^	23.7 (21.8–25.7)	23.1 (21.3–25.2)	< 0.001
Medical-aid, n (%)	172 (5.8)	51 (11.4)	< 0.001
Urban residence, n (%)	1346 (45.5)	181 (40.4)	0.050
Current smoking, n (%)	858 (29.0)	113 (25.2)	0.111
Comorbidities, n (%)			
Previous cerebrovascular accidents	476 (16.1)	113 (25.2)	< 0.001
Hypertension	1453 (49.1)	240 (53.6)	0.085
Diabetes	651 (22.0)	119 (26.6)	0.036
Dyslipidemia	275 (9.3)	25 (5.6)	0.013
Cancers	157 (5.3)	24 (5.4)	0.921
Symptomatic arrhythmias	97 (3.3)	16 (3.6)	0.855
Coronary artery diseases	49 (1.7)	5 (1.1)	0.516
Cerebral neurodegenerative diseases	152 (5.1)	27 (6.0)	0.500
Initial systolic blood pressure, mmHg	152.0 (140.0–169.0)	157.0 (140.0–177.0)	0.001
Initial diastolic blood pressure, mmHg	86.0 (78.0–96.0)	85.0 (77.0–95.0)	0.547
GUSS score	20.0 (13.0–20.0)	9.0 (7.0–14.0)	< 0.001
Arrival to initial VFSS, days	2.0 (1.0–5.0)	5.0 (3.0–8.0)	< 0.001
Stroke subtype, n (%)			
Hemorrhagic	549 (18.5)	116 (25.9)	< 0.001
Ischemic	2411 (81.5)	332 (74.1)	
Stroke territory, n (%)			
Anterior circulation	2038 (68.9)	308 (68.8)	0.313
Posterior circulation	612 (20.7)	102 (22.8)	
Combined	310 (10.5)	38 (8.5)	
Lesion side, n (%)			
Right	1267 (42.8)	206 (46.0)	0.415
Left	1388 (46.9)	201 (44.9)	
Bilateral	305 (10.3)	41 (9.1)	
Altered MS at admission, n (%)	187 (6.3)	132 (29.5)	< 0.001
Aphasia, n (%)	162 (5.5)	81 (18.1)	< 0.001
Facial asymmetry, n (%)	1184 (40.0)	284 (63.4)	< 0.001
Admission via ED, n (%)	2572 (86.9)	408 (91.1)	0.016
Initial mRS	2.0 (1.0–3.0)	3.0 (2.0–4.0)	< 0.001
Morse fall scale	35.0 (20.0–35.0)	35.0 (35.0–50.0)	< 0.001

*ED* emergency department, *GUSS* gugging swallowing screen, *mRS* modified Rankin Scale, *MS* mental status, *VFSS* videofluoroscopic swallowing study.

Comparisons of initial laboratory findings between the two groups are presented in Supplementary Table S3. The aspiration group showed a significantly lower albumin level, hemoglobin, platelet, total cholesterol, and triglyceride (*p* < 0.001, *p* = 0.012, *p* = 0.025, *p* = 0.011, and *p* < 0.001, respectively). Furthermore, the random glucose level was significantly higher in the aspiration group (*p* = 0.046).

### Aspiration screening with the GUSS

The GUSS score was significantly lower in the aspiration group (9.0 [7.0–14.0]) than in the non-aspiration group (20.0 [13.0–20.0]) (*p* < 0.001) (Table 1). When evaluating the predictive value of the GUSS for aspiration, the AUROC was 0.79 (0.77–0.81), and the cut-off score was 14.5. Based on the cut-off value, the F1 measure was 0.39, the sensitivity was 0.64, and the specificity was 0.83 (Table 2).

**Table 2:** Prediction performance.

Predictors	AUROC	F1 measure	Sensitivity	Specificity
Gugging swallowing screen	0.79 (0.77–0.81)	0.39	0.64	0.83
Ridge regression	0.81 (0.76–0.86)	0.45	0.66	0.79
Lasso regression	0.80 (0.75–0.85)	0.41	0.67	0.77
Elastic net regression	0.81 (0.76–0.86)	0.41	0.72	0.76
Random forest	0.78 (0.72–0.84)	0.30	0.24	0.96
Extreme gradient boosting	0.80 (0.75–0.85)	0.34	0.25	0.97
Support vector machines	0.66 (0.60–0.72)	0.15	0.12	0.93
*k*-nearest neighbors	0.71 (0.65–0.77)	0.32	0.66	0.67
Naïve bayes	0.78 (0.73–0.83)	0.43	0.37	0.95

*AUROC* area under the receiver operating characteristics curve.

### Machine learning models

We provide the number of samples after random allocation and target class balancing for each ML model in Supplementary Table S4. The predictive values and confusion matrix for each model are provided in Table 2 and Supplementary Table S5, respectively. Overall, the RLRs, RF, XGB, and NB algorithms showed AUROC values similar to that of the GUSS. Among the applied ML algorithms, ridge regression showed the highest AUROC (0.81 [0.76–0.86]) and F1 measure (0.45). The elastic net regression had the highest sensitivity (0.72), higher than that of the GUSS (0.64). The RF, XGB, SVM, and NB models showed low sensitivity and high specificity.

**Figure 3:**
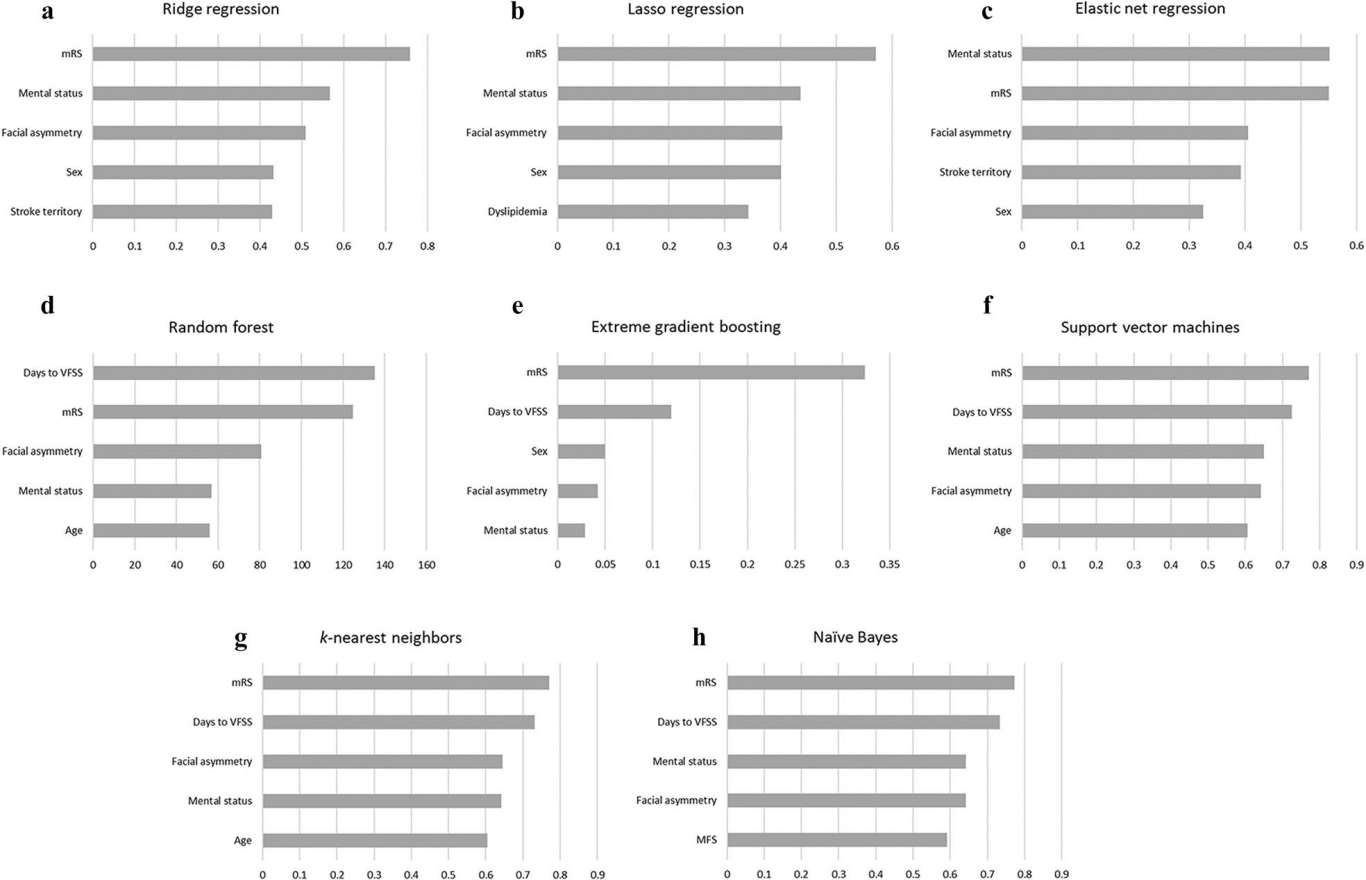
Feature importance of the optimal model: (**a**) ridge regression, (**b**) lasso regression, (**c**) elastic net regression, (**d**) random forest, (**e**) extreme gradient boosting, (**f**) support vector machines, (**g**) *k*-nearest neighbors, and (**h**) naïve Bayes. Overall, most machine learning algorithms showed similar feature importance results. The modified Rankin scale was the most important variable. Further, days to the VFSS, mental status, facial asymmetry, stroke territory, and sex were crucial features for the prediction. *MFS* Morse Fall scale, *mRS* modified Rankin Scale, *VFSS* videofluoroscopic swallowing study.

Most ML algorithms identified the modified Rankin scale as the most important variable for their performance. For RLRs, mental status, facial asymmetry, stroke territory, and sex were highly important features for the prediction. Meanwhile, days to the VFSS study were also relatively crucial for other ML algorithms’ prediction performance. The entire list of the top-five most important variables for each model is shown in Fig. 3.

### The stepwise logistic regression model

The final logistic regression model and covariates are provided in Table 3. Higher age (aOR, 1.03; 95% confidence interval [CI] 1.01–1.04; *p* < 0.001), male sex (aOR, 2.19; 95% CI 1.71–2.81; *p* < 0.001), days to initial VFSS (aOR, 1.02; 95% CI 1.01–1.04; *p* = 0.002), posterior circulation stroke (aOR, 1.59; 95% CI 1.21–2.09; *p* = 0.001), altered mental status (aOR, 2.61; 95% CI 1.89–3.60; *p* < 0.001), aphasia (aOR, 1.95; 95% CI 1.35–2.81; *p* < 0.001), higher modified Rankin scale score (aOR, 1.63; 95% CI 1.47–1.80; *p* < 0.001), previous cerebrovascular accidents (aOR, 1.48; 95% CI 1.13–1.94; *p* = 0.004), and higher systolic blood pressure (aOR, 1.11; 95% CI 1.05–1.18; *p* < 0.001) were significantly associated with a higher risk of aspiration. In contrast, facial symmetry (aOR, 0.49; 95% CI 0.39–0.62; *p* < 0.001), higher body mass index (aOR, 0.96; 95% CI 0.93–1.00; *p* = 0.039), left side lesion (aOR, 0.75; 95% CI 0.59–0.96; *p* = 0.023), and higher diastolic blood pressure (aOR, 0.87; 95% CI 0.79–0.96; *p* = 0.007) were significantly associated with a lower risk of aspiration.

**Table 3:** Final logistic regression model for identifying** r**isk factors of aspiration after acute stroke.

Variables	Adjusted OR	95% Confidence Interval	*p*-value
Age	1.03	1.01–1.04	< 0.001
Male	2.19	1.71–2.81	< 0.001
Body mass index	0.96	0.93–1.00	0.039
Previous cerebrovascular accidents	1.48	1.13–1.94	0.004
Diabetes	1.29	1.00–1.68	0.053
Cerebral neurogenerative diseases	0.62	0.38–1.01	0.054
Initial systolic blood pressure	1.11	1.05–1.18	< 0.001
Initial diastolic blood pressure	0.87	0.79–0.96	0.007
Days to initial VFSS	1.02	1.01–1.04	0.002
Stroke territory			
Anterior circulation	Reference		
Posterior circulation	1.59	1.21–2.09	0.001
Combined	0.81	0.50–1.31	0.388
Lesion side			
Right	Reference		
Left	0.75	0.59–0.96	0.023
Bilateral	0.82	0.52–1.31	0.394
Ischemic stroke	0.85	0.63–1.16	0.311
Altered MS at admission	2.61	1.89–3.60	< 0.001
Aphasia	1.95	1.35–2.81	< 0.001
Facial symmetry	0.49	0.39–0.62	< 0.001
Initial mRS	1.63	1.47–1.80	< 0.001
Blood urea nitrogen	0.99	0.97–1.00	0.120

*mRS* modified Rankin scale, *MS* mental status, *OR* odds ratio, *VFSS* videofluoroscopic swallowing study.

## Discussion

In patients hospitalized with acute stroke, we compared the predictive value of aspiration with the GUSS, an existing dysphagia screening tool. We proposed ML models based on the patients’ initial information to enable early screening. Among applied ML algorithm predictors, RLRs showed valid prediction performances and were not inferior to GUSS. This study provides a significant contribution to the field because it is the first study to develop ML models to screen aspiration in patients with acute stroke with a relatively large sample compared to related previous studies. In addition, our study demonstrated that a new aspiration screening tool could be developed by utilizing scattered and various clinical information from hospitalized patients with acute stroke. Therefore our ML models have the potential to minimize the time and efforts of medical staff for screening dysphagia after acute stroke in the clinical field and enable an efficient decision-making process, ultimately improving patient outcomes.

Early dysphagia assessment in patients with acute stroke is critical and essential for establishing a dietary and fluid intake strategy^18^. Screening tools for dysphagia can identify swallowing difficulties before confirmatory studies, such as a VFSS or fiberoptic endoscopic evaluation of swallowing, which require a separate space and scheduling. These screening tools have been found to reduce the rate of aspiration pneumonia and unnecessary tube feeding or nil per os period in patients with acute stroke^12^. However, although traditional screening tools can be applied to most patients in an awake and alert state, an accurate evaluation is impossible if there is a cognitive decline or the patient cannot obey instructions because of aphasia. In particular, questionnaires such as the dysphagia handicap index and eating assessment tool-10 are relatively more restricted by the limitations mentioned above, although they have the advantage of being non-invasive, unlike the GUSS^16,17^. Consequently, a significant limitation of these traditional screening tools is their limited ability to detect dysphagia in patients who may be highly likely at risk for aspiration conditions after acute stroke.

In this study, we demonstrated that the limitations of these existing screening tools could be overcome through ML prediction models, especially in unstable patients with acute stroke. In particular, compared to the GUSS, ML-based models demonstrated a vital advantage; they could predict aspiration with similar performance without requiring an invasive direct swallowing test. Moreover, another advantage is that aspiration screening can be performed much more readily and efficiently based on the initial clinical information obtained from patients with acute stroke^63^. Unlike traditional screening tools relying on subjective assessments by human experts, ML-based models also have the potential to screen for aspiration more objective and standardized manner by utilizing various features. Furthermore, ML models have the evolutionary potential to continuously learn and adapt to new data, leading to further improvement in their predictive performance over time. Overall, using an ML-based aspiration screening tool potentially contribute to improving patient outcomes and reducing costs.

Our newly developed ML-based screening tool showed valid performance compared to previous dysphagia screening tools. Unfortunately, few studies have attempted to predict precisely aspiration, not overall dysphagia, after acute stroke. Kim et al.^13^ identified the predictive values for aspiration on the GUSS and dysphagia handicap index in a single-center prospective study, with AUROCs of 0.77 and 0.79, respectively; our ML models’ prediction performances were not inferior to their screening tools. Warnecke et al.^11^ conducted a study to predict aspiration using the GUSS in 100 patients with acute stroke, with an AUROC confirmed as 0.76. Meanwhile, Edmiaston et al.^14^ introduced a bedside stroke dysphagia screen named Barnes-Jewish Hospital-Stroke Dysphagia Screen. In their study with 225 patients with acute stroke, sensitivity and specificity for detecting aspiration were 95% and 50%, respectively, showing relatively higher false-positive rates. Leder et al.^64^ reported clinical predictors such as dysphonia, dysarthria, abnormal gag reflex, abnormal volitional cough, cough after swallowing, and voice change after swallowing for post-stroke aspiration. Their model’s sensitivity and specificity were 80% and 30% for predicting aspiration, respectively, similar to Edmiaston et al.’s. Meanwhile, RLR models in our study showed relatively balanced sensitivity and specificity compared to other models through regularization. Consequently, the ML-based aspiration prediction models presented in this study showed similar or slightly better predictive values than the previous results of dysphagia screening tools.

This study is significant in providing clinical clues while analyzing both ML and stepwise logistic regression models; it comprehensively examined related predictors and presented their serial importance. Our results showed that functional level was a significant predictor of aspiration. The modified Rankin scale provides a functional evaluation of stroke severity^65^. Days to initial VFSS also showed high feature importance; we inferred that this feature was an indirect indicator of medical complications or functional level, demonstrating that the patient could sit upright for the VFSS. In a previous study, Henke et al.^66^ demonstrated stroke severity as a reliable and straightforward predictor, consistent with our findings. As reported in previous studies, facial asymmetry was also a significant predictor of aspiration^67,68^.

Some clinical findings showed notable results. This study showed that the dyslipidemia rate was higher in the non-aspiration group. In a previous study, Scheitz et al.^69^ reported that statin users’ risk of post-stroke pneumonia was reduced; the results of this study supported their findings, which might be related to the intravascular anti-inflammatory effect of statin^70^. However, in both groups, the frequency of dyslipidemia was less than 10%; therefore, it needs to be cautious for this interpretation. Meanwhile, in the logistic regression model of this study, it was confirmed that the higher the systolic blood pressure and the lower the diastolic blood pressure, the higher the risk of aspiration. These results were inferred because extraordinarily high or low blood pressure on admission was associated with worse stroke severity^71^. However, clinical findings such as older age, male, previous cerebrovascular disease, stroke in the posterior circulation, and altered mental status were also factors related to stroke severity. They were associated with a significantly high aspiration risk in this study, consistent with previous studies’ results^72,73^.

We designated the AUROC and F1 measures as metrics because of an imbalance in the dependent variable. A linear model slightly outperformed the ensembled algorithms and other classical classifiers utilized in our study. The best model in terms of AUROC was ridge regression. Meanwhile, the elastic net regression method, which combines ridge and lasso regularization for the linear model^74^, showed the highest sensitivity among other ML models. However, the ensembled algorithms, such as RF and XGB, showed low sensitivity with very high specificity. Thus, the ability to discriminate negative cases was high, somewhat inconsistent with the original purpose of screening aspiration. These results demonstrated that approaches using ML sometimes easily over-rely on some features, resulting in overfitting, which leads to non-generalizable results^75^. We also infer from these results that regularization methods more effectively reduced overfitting than ensemble algorithms in our dataset^76^. Appropriate regularization techniques are crucial in deriving generalizable and applicable results from ML models.

The study has several limitations. First, it was a single-center, retrospective study. Due to the study's retrospective nature, some variables related to dysphagia, such as sensory change of throat, were not included as covariates because they could not be consistently evaluated in patients with acute stroke. Moreover, the generalizability of our results is not verified and requires further validation through multicenter studies. Second, we attempted to create an ML model that can be widely applied to all patients with acute stroke. However, stroke is a broad-spectrum disease entity. If subgroup analyses may yield better model performance. Thus, future studies should establish more specific prediction models for certain stroke patients. These models can be helpful for patients with mild stroke to avoid unnecessary radiation exposure as well as enable quick decisions to prevent aspiration pneumonia in patients with severe stroke before a VFSS study. Third, we could not present longitudinal findings regarding the prediction of long-term outcomes; this limitation was primarily related to the hospital setting and rehabilitation treatment delivery system of South Korea. We observed that many patients were transferred to rehabilitation or convalescent hospitals after acute care, and some were not reliably followed long-term. Finally, we could only compare predictive values between ML algorithms and GUSS among several existing dysphagia screening tools. Future studies should verify the validity of our proposed ML models for other screening tools.

In conclusion, this study demonstrated that an ML-based screening model was not inferior to the GUSS in predicting aspiration in hospitalized patients with acute stroke. The RLRs showed better performance among the evaluated ML algorithms. Our findings suggest that ML prediction models can be efficient and straightforward, reducing the time and efforts of medical staff for dysphagia screening in patients with acute stroke. Furthermore, ML prediction models are objective and can overcome the limitations of previous dysphagia screening tools. However, additional validation is required, and specific ML models for each subgroup based on stroke severity and subtype are necessary for clinical applications.

## Supplementary Information

Supplementary Information 1.

Supplementary Information 2.

## Data Availability

All data generated or analysed during this study are included in its supplementary information files.
