# Supplementary material for: Machine learning predictive model for aspiration screening in hospitalized patients with acute stroke
Source: Sci Rep. 2023 May 15;13:7835. doi: 10.1038/s41598-023-34999-8 (PMC10185509; doi:10.1038/s41598-023-34999-8)
Supplement: Supplementary file 2 — Supplementary Information 2. [file 41598_2023_34999_MOESM2_ESM.pdf]

Supplementary Materials

Machine Learning Predictive Model for Aspiration Screening in Hospitalized Patients with Acute Stroke

Dougho Park[1,2], Seok Il Son[3], Min Sol Kim[3], Tae Yeon Kim[4], Jun Hwa Choi[5], Sang-Eok Lee[2], Daeyoung Hong[6], Mun-Chul Kim[6]

[1]Department of Medical Science and Engineering, School of Convergence Science and Technology, Pohang University of Science and Technology, Pohang, Republic of Korea

[2]Department of Rehabilitation Medicine, Pohang Stroke and Spine Hospital, Pohang, Republic of Korea

[3]Occupational Therapy Department of Rehabilitation Center, Pohang Stroke and Spine Hospital, Pohang, Republic of Korea

[4]Speech-Language Therapy Department of Rehabilitation Center, Pohang Stroke and Spine Hospital, Pohang, Republic of Korea

[5]Department of Quality Improvement, Pohang Stroke and Spine Hospital, Pohang, Republic of Korea

[6]Department of Neurosurgery, Pohang Stroke and Spine Hospital, Pohang, Republic of Korea

Corresponding author: Dougho Park, MD, PhD

Email: parkdougho@gmail.com

Supplementary Materials

Supplementary Table 1. All potential predictors and their definitions.

Supplementary Table 2. Tuned hyperparameters and searching methods for each machine learning model.

Supplementary Table 3. Initial laboratory findings.

Supplementary Table 4. The number of samples after random allocation and target class balancing for each ML model.

Supplementary Table 5. Confusion matrix.

Supplementary Document 1. R code for this study.

Supplementary Table 1. All potential predictors and their definitions.

Variables	Type	Definition
Age	Numerical	in years
Sex	Categorical	male or female
Body mass index	Numerical	measured at the time of admission; in kg/m^2
Insurance type	Categorical	medical-aid or national health insurance
Residential area	Categorical	according to the administrative distinct; urban or rural
Smoking	Categorical	current smoker or not (including quitter and non-smoker)
Comorbidities, n (%)		
Previous cerebrovascular accidents	Categorical	via patient or guardian interviews, medical records, and medication history
Hypertension	Categorical	via patient or guardian interviews, medical records, and medication history
Diabetes	Categorical	via patient or guardian interviews, medical records, and medication history
Dyslipidemia	Categorical	via patient or guardian interviews, medical records, and medication history
Cancers	Categorical	via patient or guardian interviews, medical records, and medication history
Symptomatic arrhythmias	Categorical	via patient or guardian interviews, medical records, and medication history
Coronary artery diseases	Categorical	via patient or guardian interviews, medical records, and medication history
Cerebral neurogenerative diseases	Categorical	via patient or guardian interviews, medical records, and medication history
Initial systolic blood pressure	Numerical	measured at the time of admission; in mmHg
Initial diastolic blood pressure	Numerical	measured at the time of admission; in mmHg
Arrival to initial VFSS	Numerical	days from admission to initial VFSS
Stroke subtype		
Hemorrhagic	Categorical	ICD-10 code of I60, I61, and I62
Ischemic	Categorical	ICD-10 code of I63
Stroke territory		
Anterior circulation	Categorical	ACA and MCA territories
Posterior circulation	Categorical	VA, BA, and PCA territories
Combined	Categorical	both anterior and posterior
Lesion side		
Right	Categorical	lesion localized in the right side
Left	Categorical	lesion localized in the left side
Bilateral	Categorical	multiple lesions in bilateral sides; lesion beyond the midline
Altered MS at admission	Categorical	alert or not (including drowsy, confusion, semi-coma, and coma status)
Aphasia	Categorical	presence of all kinds of aphasia (motor, sensory, global, and anomic) or not

Facial asymmetry	Categorical	via inspection; identification of dysarthria
Admission via ED	Categorical	ED or outpatient clinic
Initial mRS	Numerical	measured by a neurosurgeon, neurologist, or rehabilitation specialist within 48 hours after the admission
Morse Fall scale	Numerical	measured by a nursing department within 48 hours after the admission
Albumin	Numerical	measured at the time of admission; in g/dl
Random glucose	Numerical	measured at the time of admission; in mg/dl
Blood urea nitrogen	Numerical	measured at the time of admission; in mg/dl
Creatinine	Numerical	measured at the time of admission; in mg/dl
Hemoglobin	Numerical	measured at the time of admission; in g/dl
Platelets	Numerical	measured at the time of admission; in $10^3/\mu L$
Total cholesterol	Numerical	measured within 48 hours after the admission; in mg/dl
High density lipoprotein	Numerical	measured within 48 hours after the admission; in mg/dl
Low density lipoprotein	Numerical	measured within 48 hours after the admission; in mg/dl
Triglycerides	Numerical	measured within 48 hours after the admission; in mg/dl

Abbreviations: ACA, anterior cerebral artery; BA, basilar artery; ED, emergency department; ICD, international classification of diseases; MCA, middle cerebral artery; mRS, modified Rankin scale; PCA, posterior cerebral artery; VA, vertebral artery; VFSS, videofluoroscopic swallowing study.

Supplementary Table 2. Tuned hyperparameters and searching methods for each machine learning model.

Predictors	Searching methods	Optimal hyperparameters for each model
Ridge regression	Grid	alpha = 0; lamda = 0.044
Lasso regression	Grid	alpha = 1; lamda = 0.044
Elastic net regression	Grid	alpha = 0.5; lamda = 0.043
Random forest	Random	ntree = 500; mtry = 2
Extreme gradient boosting	Grid	nrounds = 1500; max_depth = 8; eta = 0.01; gamma = 0; colsample_bytree = 1; min_child_weight = 1; subsample =1
Support vector machines	Random	sigma = 0.012; C = 64
k-nearest neighbors	Random	k = 5
Naïve Bayes	Random	fL = 0; usekernel = TRUE; adjust = 1.0

Supplementary Table 3. Initial laboratory findings.

Variables	No aspiration (n = 2960)	Aspiration (n = 448)	*p*-value
Albumin, g/dl	4.2 (4.0–4.4)	4.1 (3.9–4.3)	<0.001
Random glucose, mg/dl	128.0 (108.0–163.0)	132.0 (110.5–169.0)	0.046
Blood urea nitrogen, mg/dl	15.5 (12.6–19.4)	15.6 (12.5–19.9)	0.750
Creatinine, mg/dl	0.8 (0.6–0.9)	0.8 (0.6–1.0)	0.104
Hemoglobin, g/dl	13.9 (12.6–15.1)	13.6 (12.4–14.7)	0.012
Platelet, $10^3/\mu L$	235.0 (196.0–277.0)	226.5 (182.0–272.0)	0.025
Total cholesterol, mg/dl	186.0 (156.0–218.0)	179.0 (150.0–210.0)	0.011
High density lipoprotein, mg/dl	48.0 (40.0–56.0)	47.5 (40.0–55.0)	0.775
Low density lipoprotein, mg/dl	106.8 (78.2–134.3)	104.0 (76.0–132.8)	0.306
Triglyceride, mg/dl	129.0 (92.0–187.0)	117.0 (81.0–167.0)	<0.001

Supplementary Table 4. The number of samples after random allocation and target class balancing for each ML model.

Predictors	Training set		Training set after SMOTE		Test set	
	No-ASP	ASP	No-ASP	ASP	No-ASP	ASP
Ridge regression	2247	330	2247	1980	713	118
Lasso regression	2216	338	2216	2028	744	110
Elastic net regression	2211	351	2211	2106	749	97
Random forest	2173	359	2173	2154	787	89
Extreme gradient boosting	2219	340	2219	2040	741	108
Support vector machines	2249	342	2240	2106	720	97
k-nearest neighbors	2240	340	2240	2106	720	97
Naïve Bayes	2229	336	2229	2106	731	112

Abbreviations: ASP, aspiration; SMOTE, synthetic minority oversampling technique.

Supplementary Table 5. Confusion matrix.

**Predictors**	**Prediction**	**Reference**	
		No-ASP	ASP
Gugging Swallowing Screen	No-ASP	1896	77
	ASP	1064	371
Ridge regression	No-ASP	563	40
	ASP	150	78
Lasso regression	No-ASP	569	36
	ASP	175	74
Elastic net regression	No-ASP	570	27
	ASP	179	70
Random forest	No-ASP	757	68
	ASP	30	21
Extreme gradient boosting	No-ASP	719	81
	ASP	22	27
Support vector machines	No-ASP	672	85
	ASP	48	12
k-nearest neighbors	No-ASP	479	33
	ASP	241	64
Naïve Bayes	No-ASP	694	71
	ASP	37	41

Abbreviation: ASP, aspiration.

Supplementary Document 1. R code for this study.

```
library(readr)
library(caret)
library(caretEnsemble)
library(data.table)
library(dplyr)
library(tidyverse)
library(LiblineaR)
library(randomForest)
library(xgboost)
library(e1071)
library(gbm)
library(kernlab)
library(smotefamily)
library(GGally)
library(MLeval)
library(moonBook)
library(pROC)
library(doParallel)
library(klaR)
library(caTools)
library(mice)
library(glmnet)

ASP<- read_csv("stepwise.csv")

ASP$class<-as.factor(ASP$class)
ASP$sex<-as.factor(ASP$sex)
ASP$insur<-as.factor(ASP$insur)
ASP$resid<-as.factor(ASP$resid)
ASP$type<-as.factor(ASP$type)
ASP$side<-as.factor(ASP$side)
ASP$territory<-as.factor(ASP$territory)
ASP$route<-as.factor(ASP$route)
ASP$ini_ms<-as.factor(ASP$ini_ms)
ASP$aphasia<-as.factor(ASP$aphasia)
ASP$facial<-as.factor(ASP$facial)
ASP$cva<-as.factor(ASP$cva)
ASP$htn<-as.factor(ASP$htn)
ASP$dia<-as.factor(ASP$dia)
ASP$sm<-as.factor(ASP$sm)
ASP$dyslip<-as.factor(ASP$dyslip)
ASP$cancer<-as.factor(ASP$cancer)
ASP$arr<-as.factor(ASP$arr)
ASP$cad<-as.factor(ASP$cad)
ASP$nd<-as.factor(ASP$nd)


ASP1<-na.omit(ASP)
ASP1$guss=NULL
```

```
model1<-glm(class~., data=ASP1, family=binomial)
reduced.model1=step(model1)



model2<-glm(class~vfss_days+age+BMI+sbp10+dbp10+bun+mrs+sex+type+side+terr
itory+ini_ms+aphasia+facial+cva+dia + nd, data=ASP1, family=binomial)

extractOR(model2)
vif(model2)
sqrt(vif(model2))>2


GUSS <- roc (ASP$class, ASP$guss)
plot.roc(GUSS, print.auc=TRUE, max.auc.polygon=TRUE, print.thres=TRUE, pri
nt.thres.pch=19, print.thres.col = "red", auc.polygon=TRUE)

mytable(class~., data=ASP, method=3)

colSums(is.na(ASP))

miceresults<-mice(ASP, seed=1234, m=5)

complete_data_reg<-complete(miceresults,1)
write.csv(complete_data_reg,file="complete_data_reg.csv")
predata<-complete_data_reg

predata$guss=NULL

nearZeroVar(predata)

predata$arr=NULL
predata$cad=NULL
predata$nd=NULL

ggpairs(predata[,2:18], lower=list(continuous="smooth"))

st_model<-preProcess(predata[,2:18], method=c("center","scale"))

data<-predict(st_model, predata)
data=as.data.frame(data)
ohe_feats=c('sex','insur','resid','type','side','territory','route','ini_m
s','aphasia','facial','cva','htn','dia','sm','dyslip','cancer')
dummies=dummyVars(~sex+insur+resid+type+side+territory+route+ini_ms+aphasi
a+facial+cva+htn+dia+sm+dyslip+cancer, data = data)
df_ohe <- as.data.frame(predict(dummies, newdata = data))
df_combined <- cbind(data[,-c(which(colnames(data) %in% ohe_feats))],df_oh
e)
dat = as.data.table(df_combined)

table(dat$class)

seed<-1234
set.seed(seed)
ind<-sample(2,nrow(dat),replace = T,prob = c(0.75,0.25))
traindata<-dat[ind==1,]
```

```r
testdata<-dat[ind==2,]

set.seed(seed)
traindata.smote <- SMOTE(traindata[,-1], traindata$class ,K = 5, dup_size=
0)
traindata.smote <- traindata.smote$data
traindata.smote$class <- as.factor(traindata.smote$class)
table(traindata.smote$class)

traindata.smote$Newclass<-relevel(traindata.smote$class, ref = "N")
levels(traindata.smote$Newclass)

table(traindata.smote$Newclass)

traindata.smote$class=NULL

detectCores()

cl <- makePSOCKcluster(12)
registerDoParallel(cl)

ctrl <- trainControl(method="repeatedcv", number=5, repeats = 50, allowPar
allel=TRUE)

set.seed(seed)
ridge <- train(Newclass~., data = traindata.smote, method = "glmnet", tune
grid=data.frame(alpha=0, lqmda=seq(0, 0.3,by=0.05)), verbose=FALSE)

pred_lr<- predict(ridge, newdata=testdata)
confusionMatrix(pred_lr, testdata$class)

pred_bagLR <- predict(ridge, newdata = testdata, type = "prob")[, "Y"]
pred_bagLR <- data.frame(pred_bagLR)
caTools::colAUC(pred_bagLR, testdata$class, plotROC = TRUE)

test_prob_smotelr <- predict(ridge, newdata = testdata, type="prob")
lrsmote = data.frame(test_prob_smotelr, testdata$class)
lrsmote<-evalm(lrsmote)

lrsmote$stdres

imp_lrsmote<-varImp(ridge, scale = FALSE)
imp_lrsmote

seed<-1111
set.seed(seed)

ind<-sample(2,nrow(dat),replace = T,prob = c(0.75,0.25))
traindata<-dat[ind==1,]
testdata<-dat[ind==2,]

set.seed(seed)
traindata.smote <- SMOTE(traindata[,-1], traindata$class ,K = 5, dup_size=
0)
traindata.smote <- traindata.smote$data
```

```
traindata.smote$class <- as.factor(traindata.smote$class)
table(traindata.smote$class)

traindata.smote$Newclass<-relevel(traindata.smote$class, ref = "N")
levels(traindata.smote$Newclass)

table(traindata.smote$Newclass)

traindata.smote$class=NULL

set.seed(seed)
lasso <- train(Newclass~., data = traindata.smote, method = "glmnet", tune
grid=data.frame(alpha=1, lqmda=seq(0, 0.3,by=0.05)), verbose=FALSE)

pred_lr<- predict(lasso, newdata=testdata)
confusionMatrix(pred_lr, testdata$class)

pred_bagLR <- predict(lasso, newdata = testdata, type = "prob")[, "Y"]
pred_bagLR <- data.frame(pred_bagLR)
caTools::colAUC(pred_bagLR, testdata$class, plotROC = TRUE)

test_prob_smotelr <- predict(lasso, newdata = testdata, type="prob")
lrsmote = data.frame(test_prob_smotelr, testdata$class)
lrsmote<-evalm(lrsmote)

lrsmote$stdres

imp_lrsmote<-varImp(lasso, scale = FALSE)
imp_lrsmote

seed<-1211
set.seed(seed)

ind<-sample(2,nrow(dat),replace = T,prob = c(0.75,0.25))
traindata<-dat[ind==1,]
testdata<-dat[ind==2,]

set.seed(seed)
traindata.smote <- SMOTE(traindata[,-1], traindata$class ,K = 5, dup_size=
0)
traindata.smote <- traindata.smote$data
traindata.smote$class <- as.factor(traindata.smote$class)
table(traindata.smote$class)

traindata.smote$Newclass<-relevel(traindata.smote$class, ref = "N")
levels(traindata.smote$Newclass)

table(traindata.smote$Newclass)

traindata.smote$class=NULL

set.seed(seed)
elastic <- train(Newclass~., data = traindata.smote, method = "glmnet", tu
negrid=data.frame(alpha=1, lqmda=seq(0, 0.3,by=0.05)), verbose=FALSE)
```

```
pred_lr<- predict(elastic, newdata=testdata)
confusionMatrix(pred_lr, testdata$class)

pred_bagLR <- predict(elastic, newdata = testdata, type = "prob")[, "Y"]
pred_bagLR <- data.frame(pred_bagLR)
caTools::colAUC(pred_bagLR, testdata$class, plotROC = TRUE)

test_prob_smotelr <- predict(elastic, newdata = testdata, type="prob")
lrsmote = data.frame(test_prob_smotelr, testdata$class)
lrsmote<-evalm(lrsmote)

lrsmote$stdres

imp_lrsmote<-varImp(elastic, scale = FALSE)
imp_lrsmote

ctrl <- trainControl(method="repeatedcv", number=5, repeats = 50, classPro
bs = TRUE, allowParallel=TRUE)
metric <- "ROC"

seed<-1123
set.seed(seed)

ind<-sample(2,nrow(dat),replace = T,prob = c(0.75,0.25))
traindata<-dat[ind==1,]
testdata<-dat[ind==2,]

set.seed(seed)
traindata.smote <- SMOTE(traindata[,-1], traindata$class ,K = 5, dup_size=
0)
traindata.smote <- traindata.smote$data
traindata.smote$class <- as.factor(traindata.smote$class)
table(traindata.smote$class)

traindata.smote$Newclass<-relevel(traindata.smote$class, ref = "N")
levels(traindata.smote$Newclass)

table(traindata.smote$Newclass)

traindata.smote$class=NULL

set.seed(seed)
svm_fit_smote <- train(Newclass~., data = traindata.smote, method = "svmRa
dial", trControl=ctrl, metric=metric, tuneLength = 10)
svm_fit_smote

test_pred_svm <- predict(svm_fit_smote, newdata = testdata)
confusionMatrix (test_pred_svm, testdata$class)

test_prob_svm <- predict(svm_fit_smote, newdata = testdata, type="prob")
msvm = data.frame(test_prob_svm, testdata$class)
ysvm<-evalm(msvm)

ysvm$stdres
```

```
imp_svmsmote<-varImp(svm_fit_smote, scale = FALSE)
imp_svmsmote

seed<-1123
set.seed(seed)

ind<-sample(2,nrow(dat),replace = T,prob = c(0.75,0.25))
traindata<-dat[ind==1,]
testdata<-dat[ind==2,]

set.seed(seed)
traindata.smote <- SMOTE(traindata[,-1], traindata$class ,K = 5, dup_size=
0)
traindata.smote <- traindata.smote$data
traindata.smote$class <- as.factor(traindata.smote$class)
table(traindata.smote$class)

traindata.smote$Newclass<-relevel(traindata.smote$class, ref = "N")
levels(traindata.smote$Newclass)

table(traindata.smote$Newclass)

##
##    N    Y
## 2240 2106

traindata.smote$class=NULL

set.seed(seed)
knn_fit_smote <- train(Newclass~., data = traindata.smote, method = "knn",
 trControl=ctrl, metric=metric, tuneLength = 10)
knn_fit_smote

test_pred_knn <- predict(knn_fit_smote, newdata = testdata)
confusionMatrix (test_pred_knn, testdata$class)

test_prob_knn <- predict(knn_fit_smote, newdata = testdata, type="prob")
mknn = data.frame(test_prob_knn, testdata$class)
yknn<-evalm(mknn)

yknn$stdres

imp_knnsmote<-varImp(knn_fit_smote, scale = FALSE)
imp_knnsmote

seed<-1112
set.seed(seed)

ind<-sample(2,nrow(dat),replace = T,prob = c(0.75,0.25))
traindata<-dat[ind==1,]
testdata<-dat[ind==2,]

set.seed(seed)
traindata.smote <- SMOTE(traindata[,-1], traindata$class ,K = 5, dup_size=
0)
```

```
traindata.smote <- traindata.smote$data
traindata.smote$class <- as.factor(traindata.smote$class)
table(traindata.smote$class)

traindata.smote$Newclass<-relevel(traindata.smote$class, ref = "N")
levels(traindata.smote$Newclass)

table(traindata.smote$Newclass)

traindata.smote$class=NULL

set.seed(seed)
nb_fit_smote <- train(Newclass~., data = traindata.smote, method = "nb", t
rControl=ctrl, metric=metric, tuneLength = 10)
nb_fit_smote

test_pred_nb <- predict(nb_fit_smote, newdata = testdata)
confusionMatrix (test_pred_nb, testdata$class)

test_prob_nb <- predict(nb_fit_smote, newdata = testdata, type="prob")
mnb = data.frame(test_prob_nb, testdata$class)
ynb<-evalm(mnb)

ynb$stdres

imp_nbsmote<-varImp(nb_fit_smote, scale = FALSE)
imp_nbsmote

seed<-0923
set.seed(seed)

ind<-sample(2,nrow(dat),replace = T,prob = c(0.75,0.25))
traindata<-dat[ind==1,]
testdata<-dat[ind==2,]

set.seed(seed)
traindata.smote <- SMOTE(traindata[,-1], traindata$class ,K = 5, dup_size=
0)
traindata.smote <- traindata.smote$data
traindata.smote$class <- as.factor(traindata.smote$class)
table(traindata.smote$class)

traindata.smote$Newclass<-relevel(traindata.smote$class, ref = "N")
levels(traindata.smote$Newclass)

table(traindata.smote$Newclass)

traindata.smote$class=NULL

set.seed(seed)
fit.rf <- train(Newclass~., data = traindata.smote, method = "rf", metric
= metric, trControl = ctrl, tuneLength=10)
fit.rf

pred_rf<- predict(fit.rf, newdata=testdata)
confusionMatrix(pred_rf, testdata$class)
```

```
test_prob_smoterf <- predict(fit.rf, newdata = testdata, type="prob")
rfsmote = data.frame(test_prob_smoterf, testdata$class)
rfsmote<-evalm(rfsmote)

rfsmote$stdres

imp_rfsmote<-varImp(fit.rf, scale = FALSE)
imp_rfsmote

seed<-0105
set.seed(seed)

ind<-sample(2,nrow(dat),replace = T,prob = c(0.75,0.25))
traindata<-dat[ind==1,]
testdata<-dat[ind==2,]

set.seed(seed)
traindata.smote <- SMOTE(traindata[,-1], traindata$class ,K = 5, dup_size=
0)
traindata.smote <- traindata.smote$data
traindata.smote$class <- as.factor(traindata.smote$class)
table(traindata.smote$class)

traindata.smote$Newclass<-relevel(traindata.smote$class, ref = "N")
levels(traindata.smote$Newclass)

table(traindata.smote$Newclass)

traindata.smote$class=NULL

tune_grid <- expand.grid(
  nrounds = c(1000, 1500, 2000),
  eta = c(0.01, 0.1, 0.3),
  max_depth = c(6, 7, 8),
  gamma = 0,
  colsample_bytree = 1,
  min_child_weight = 1,
  subsample = 1
)

set.seed(seed)
fit.xgb <- train(Newclass~., data = traindata.smote, method = "xgbTree", m
etric = metric, trControl = ctrl, tuneGrid = tune_grid)
fit.xgb

pred_xgb<- predict(fit.xgb, newdata=testdata)
confusionMatrix(pred_xgb, testdata$class)

test_prob_smotexgb <- predict(fit.xgb, newdata = testdata, type="prob")
xgbsmote = data.frame(test_prob_smotexgb, testdata$class)
xgbsmote<-evalm(xgbsmote)

xgbsmote$stdres

imp_xgbsmote<-varImp(fit.xgb, scale = FALSE)
imp_xgbsmote
```
